# Supplementary material for: Adoption of policies to improve respectful maternity care in Timor-Leste
Source: PLoS One. 2024 Mar 25;19(3):e0289394. doi: 10.1371/journal.pone.0289394 (PMC10962841; doi:10.1371/journal.pone.0289394)
Supplement: S1 Table — (DOCX) [file pone.0289394.s001.docx]

**S1 Table Quality Measures for Experience of Care**

|  |
| --- |

| **No** | **Quality measures** | | **Health facility 1** | **Health facility 2** | | **Health facility 3** | |
| --- | --- | --- | --- | --- | --- | --- | --- |
| **Quality Measures for Effective Communication** | | | | | | | |
| 1 | Health education materials are available in Tetum, with easily accessible writing or pictures, and are given by midwives to birthing women. | | No | No | | No | |
| 2 | Midwives are oriented and receive in-service training at least once every 12 months to improve communication and interpersonal counselling skills according to local culture. | | No | No | | No | |
| 3 | The maternity department has a written, up-to-date policy that outlines clear goals, operational plans and monitoring mechanisms to promote the interpersonal communication and counselling skills of midwives. | | No | No | | No | |
| 4 | Midwives in the maternity unit receive supportive supervision in interpersonal communication, counselling and cultural competence every three months. | | No | No | | No | |
| **Quality Measures for Privacy and Confidentiality** | | | | | | | |
| 1 | The physical environment of the health facility allows for privacy and respectful, confidential care, including the availability of curtains, screens, partitions and adequate bed capacity. | Yes | | | Yes | | No |
| 2 | The health facility has written and up-to-date protocols to ensure privacy and confidentiality for all women during birth. | No | | | No | | No |
| **Quality Measures to Ensure no Woman is Subjected to Mistreatment** | | | | | | | |
| 1 | The health facility has written, up-to-date, zero-tolerance non-discriminatory policies with regard to the mistreatment of women in the maternity ward. | No | | | No | | No |
| 2 | The health facility has a written accountability mechanism in the event of mistreatment or violence | No | | | No | | No |
| 3 | The health facility has up-to-date written policies and protocols outlining the rights of women to make complaints about the care received and has an easily accessible mechanism (eg, comments box) for submitting complaints. | No | | | No | | No |
| 4 | Midwives receive in-service training and supportive supervision in respecting the rights of women, and providing respectful care. Orientation is given to new staff. | No | | | No | | No |
| 5 | The health facility has a complaints box, which is easily accessible to women and their families, which is periodically emptied and the contents reviewed. | No | | | No | | No |
| **Quality Measures for Informed Choice** | | | | | | | |
| 1 | The health facility has a written and up-to-date policy for obtaining the consent of the woman prior to examinations and procedures. | No | | | No | | No |
| 2 | Health facilities have standard informed consent forms that help midwives provide women with easy-to-understand information to obtain full consent before taking action | No | | | No | | No |
| 3 | Midwives in the maternity unit receive in-service training and supportive supervision in informed consent and women’s right to choose their care. Orientation is provided for new staff. | No | | | No | | No |
| **Quality Measures for Companion of Choice** | | | | | | | |
| 1 | The labour and childbirth areas are organized in such a way as to allow a physical private space for the woman and her companion at the time of birth. | Yes | | | Yes | | No |
| 2 | The health facility has a written, up-to-date protocol, which is explained to women and their families, to encourage all women to have at least one person of their choice, as culturally appropriate, with them during labour, childbirth and the immediate postnatal period. | No | | | No | | No |
| 3 | Midwives in the health facility are oriented and receive in-service refresher training sessions at least once every 12 months on the evidence for and positive impact of the presence of a chosen companion during labour and birth. | No | | | No | | No |
| 4 | Orientation sessions and information (written or pictorial) are available to orient the companion on his or her role in supporting the woman during labour and birth. | No | | | No | | No |
| **Quality Measures to Strengthen Women’s Capability** | | | | | | | |
| 1 | Midwives in the labour and childbirth areas of the maternity unity were oriented in nonpharmacological and pharmacological pain relief and received in-service training or sessions at least once in the preceding 12 months. | No | | | No | | No |
| 2 | The health facility has a written, up-to-date protocol, which is explained to women and their families, to minimise unnecessary interventions, support normal labour and strengthen the woman’s capability, so that she feels in control of her childbirth experience. | No | | | No | | No |
| 3 | Midwives in the labour and childbirth areas of the maternity unit were oriented and received in-service training or refresher sessions at least once in the preceding 12 months to strengthen their interpersonal and cultural competence in providing emotional support. | No | | | No | | No |
