# Supplementary material for: Adoption of policies to improve respectful maternity care in Timor-Leste
Source: PLoS One. 2024 Mar 25;19(3):e0289394. doi: 10.1371/journal.pone.0289394 (PMC10962841; doi:10.1371/journal.pone.0289394)
Supplement: Attachment — Submitted filename: Respectful maternity care_Response to reviewers_4 Dec 2023.docx [file pone.0289394.s002.docx]

# Respectful maternity care

# Response to reviewers

Dear editor,

Thank you for the opportunity to revise the manuscript ‘Adoption of policies to improve respectful maternity care in Timor-Leste’. We have made the suggested changes and responded to each of the comments below.

Kind regards,

Angelina Fernandes

***Journal requirements***

1. Please ensure that your manuscript meets PLOS ONE's style requirements, including those for file naming.

**Response: We have re-named the files according to the guidelines.**

2. We note that you have provided additional information within the Acknowledgements Section that is not currently declared in your Funding Statement. Please note that funding information should not appear in the Acknowledgments section or other areas of your manuscript. We will only publish funding information present in the Funding Statement section of the online submission form. Please remove any funding-related text from the manuscript and let us know how you would like to update your Funding Statement.

**Response: The following sentence has been removed from the acknowledgements section in the manuscript “This research was funded through AF’s PhD scholarship by Airlangga University, Indonesia and Instituto Superior Cristal, Timor-Leste.**

3. Please state what role the funders took in the study. If the funders had no role, please state: "The funders had no role in study design, data collection and analysis, decision to publish, or preparation of the manuscript." Please include this amended Role of Funder statement in your cover letter; we will change the online submission form on your behalf.

**Response: The following sentence should be added to the online submission “This research was funded through AF’s PhD scholarship by Airlangga University, Indonesia and Instituto Superior Cristal, Timor-Leste. The funders had no role in study design, data collection and analysis, decision to publish, or preparation of the manuscript.”**

4. Upon re-submitting your revised manuscript, please upload your study’s minimal underlying data set as either Supporting Information files or to a stable, public repository and include the relevant URLs, DOIs, or accession numbers within your revised cover letter.

**Response: The raw data tables are already presented within the manuscript. We have also attached as a separate file under supporting information.**

5. Please include captions for your Supporting Information files at the end of your manuscript, and update any in-text citations to match accordingly. Please see our Supporting Information guidelines for more information: <http://journals.plos.org/plosone/s/supporting-information>

**Response: We have included captions for the supporting files and made reference to them within the text to match (Line 127 and 137).**

6. Please review your reference list to ensure that it is complete and correct.

**Response: We have reviewed and amended the references list.**

***Reviewer #1:***

• WHO standards and quality statements (4-6 on experience of care) need elaboration. For example, what is 4-6 on experience of care? Briefly introduce the WHO standards from one to N and why the 4-6 is chosen. The author/s must introduce the standards appropriately.

**Response: We have outlined in more detail the 8 standards in the WHO framework, and linked it more clearly to the importance of focusing on the domain of ‘experiences of care’. See lines 56-75.**

• The questions pertaining to ‘input quality measures’ at the facilities were recorded in a tick box column; ‘yes’ or ‘no’ type. The Likert scale method could have been more appropriate.

**Response: The guidelines, protocols and equipment are either present or absent within the health facilities, therefore the appropriate measure is yes/no, not a continuous variable.**

• What are the “four dot points” in the Timor-Leste guidelines?

**Response: We have clarified the dot points as they appear in the Timor-Leste guidelines, and further illustrated how they relate to the WHO standards of care (line 166-187).**

• In the introduction, with the use of data, it is shown that non-institutional deliveries increase with second and third childbirths; was this phenomenon explored through the qualitative interviews? If yes, what were the gender or power dynamics in that behaviour?

**Response: This research was designed as an assessment of policy, guidelines and procedures. The sociological dynamics of where women give birth and the power dynamics surrounding that are beyond the scope of this paper. We have, however, referenced a paper that has been published on women’s birth choices in Timor-Leste and the individual, family and social dimensions that influence place of birth (Line 83).**

***Reviewer #2:***

The overall paper underlines the importance of policy discourse and its influence on healthcare practices. It rightly calls for a fundamental shift in the culture of care for women in Timor-Leste and suggests a two-pronged approach involving immediate actions and long-term strategies. This paper provides a logical and actionable way forward based on the study's findings.

**Response: Thank you for acknowledging the importance of this work.**

The paper does not mention any potential limitations of the study, such as the scope, sample size, or potential biases. Including this information would offer a more complete understanding of the research's boundaries.

**Response: Limitations have been added at the end of the methods (Line 153-159).**

Additionally, it does not explicitly state whether this is a qualitative or quantitative study, which could be important for readers seeking specific research methods.

**Response: We have clarified that this is a mixed methods study consisting of qualitative review of national policy (deductive content analysis) and quantitative survey of health facilities (Line 96-97).**
